# Dr. Bright's Report's of Medical Cases, Illustrating the Symptoms and Cure of Diseases

**Published:** 1832-01-01

**Authors:** 


### Medico-Chirurgical Review,

### No. XXXI.

OCTOBER 1, 1831, TO JANUARY 1, 1832.

### I.

REPORTS OF MEDICAL CASES, SELECTED WITH A VIEW OF ILLUSTRATING THE SYMPTOMS AND CURE OF DISEASES, BY A REFERENCE TO MORBID ANATOMY. By *Richard Bright*, M.D. F.R.S. &c. Vol. II. London, 1831.

[Second Article.]

A FEW facts, hastily noticed and imperfectly concocted, have often been worked up into a stupendous fabric, upon which the whole system of medical science has been made to repose; and not seldom has it happened, that laborious codes of therapeutic principles have been spun out of some seducing speculations of the brain, which have neither been supported by ordinary experience, nor even countenanced by an ordinary share of plausibility. Although, therefore, to some it may be the most tedious, it is the most faithful system of therapeutics which is extracted from a well-digested selection of cases. The symptoms of even the most uniform disease so extremely fluctuate, and are checquered by so many modifying causes, that to group within a few lines its pathognomonic features is no ordinary task, and to particularize all its varying lights and shades is utterly impracticable. The general outlines are, no doubt, easily learned, because easily seen; but the finer pencilling requires taste to be discerned, and when discerned requires judgment to make it practically useful.

Every practitioner, therefore, who aims at excellence, will have but little respect for nosological arrangements, distinctions and definitions. As soon as he has opportunity he will read disease with his own eyes out of the orginal; he will trace at the bedside of sickness, with his own pencil, the devious workings of disordered nature; he will court personal familiarity with every morbid appearance; he will pursue the relations between external signs and internal states through all their labyrinths of intricacy; in fine, he will study the capricious aberrations of disease from the prescribed and general rules, as attentively as her ordinary observance of the general rules themselves, and he will neither rest contented with the model etchings of another's pencil, nor with the dictatorial delineations of any established school.

This, no doubt, requires both patience and opportunity; an undivided love for the advancement of the profession and an unreserved appropriation of the whole mind to its cultivation; but there can be only one excuse for forming our faith out of the creed of others—when we dare not confide in our own judgment, or our own honesty, in constructing a system for ourselves. To look with our own eyes is more laborious than to see through the eyes of our predecessors, and to test statements, systems and authorities at the fountain from which they were themselves taken, may only tell into the treasury after a course of time; but the experience of upwards of three thousand years has now shown that "verba magistri jurando" has much more devotion to recommend it than either industry or judgment. The child, who feels strength sufficient to go forth without support, is only tripped and trammelled by the walking chair; and the sooner that the school-books of a medical practitioner's education are abandoned, after the volume of nature has been fully submitted to his own inspection, the more rapidly will he advance in the true knowledge of disease, and the more faithfully will he be enabled to meet the high responsibilities of his official station. Hippocrates did more for medicine in thirty years than all his successors down to Haller; and when we examine his works and analyze the spirit with which they were constructed, no adequate reason can be found to account for this extraordinary disproportion of success, but his cautious observance of nature and his uniform dread of theory.

In our former article on the Work before us, we brought before our readers Dr. Bright's observations on the *Inflammatory* affections of the head; including erysipelas of the scalp and face, inflammation and suppuration of the ear, arachnitis, delirium tremens, acute and chronic hydrocephalus, inflammation and ulceration of the cerebral substance, softening of the brain, and, finally, the effects of cerebral pressure from vascular turgescence. Vascular turgescence is, however, only one cause of *Cerebral Pressure,* and one which can in general be more easily removed, if treatment be had recourse to in proper time, than any other. Depression of the skull-cap, tumors in the substance of the brain, and effusion upon its surface, or within its ventricles, in common with other causes, very frequently produce symptoms of pressure. This effusion may be of serum, or of red blood. When of serum, it may exist between the membranes, or within the ventricles, or in both situations; and it may arise from many causes, but in every instance it is probable that where actual inflammation did not previously exist, more or less vascular turgescence has preceded it. Very marked vascularity of either the membranes or substance of the brain is seldom found accompanied by great serous effusion. These states very rarely coexist, and this fact alone is strongly corroborative of the doctrine, that they stand in some etiological relation to each other. Constitutional, or topical debility has, no doubt, been occasionally observed during life, in cases where a large quantity of serous fluid has been discovered in the brain by dissection; and a pure hydropical diathesis may invade the exhalents of the sensorial structure, as well as any other branch of that system; but while every observant pathologist will admit that such cases are not only rare, but frequently complicated by symptoms of equivocal import, debility itself even in such cases cannot be regarded in any other light than as one cause of congestion. After a vessel has lost its natural tone, it can accommodate more fluid while it can propel less, and, therefore, so long as this state continues, and the circulation in other parts of the body maintains its equilibrium, the texture, which this debilitated vessel supplies, must receive more than its proportion of the general mass of blood. The Doctor has already recorded several causes of serous effusion, such as inflammation of the integuments of the head, arachnitis, cerebral sympathy with diseased organs distantly situated, inflammation of the substance of the brain, tumors within the cerebrum and vascular turgescence. In the cases, which follow, other causes of this morbid state are adduced; but they are all obviously connected with a congestive state. In the first case, which shall be extracted, direct mechanical pressure had been applied, and effusion followed the impediment, which was thus thrown in the way of the venous circulation. In five other instances effusion arose from the respiration of carbonaceous vapours. In the 109th history diseased heart was the exciting cause. And, lastly, several interesting cases of the effects of diseased kidneys upon the cerebral circulation are recorded. No doubt other causes of effusion within the head could have been brought forward; such as the action of extreme cold, submersion and excessive hæmorrhage; and—

"In some cases (says the Dr.) simple debility is perhaps capable of producing this effect, of which phthisis and diabetes seem occasionally to afford examples; though in both of these, other causes are brought into action, besides debility, in a manner likely to favour the effusion. We have sometimes less questionable examples of this cause of serous effusion, afforded in hæmorrhagic diseases and in the exsanguine constitutions of anasarcous patients, though in a great majority of these cases decided affections of the kidney, or of some other important organ, as the spleen, serve to throw doubt upon the extent to which debility should be considered the prevailing cause of the effusion." 265.

(103.) The effects of suspension by the cord are seen in Esther Hibner, whose body was inspected 48 hours after execution. The inside of the skull-cap was mottled with vascularity, the small vessels of the dura mater were turgid with blood, the longitudinal sinus was almost empty, but the other sinuses were full. The arachnoid and pia mater were not remarkable for vascularity, but between these membranes there was a considerable quantity of effused fluid, and the ventricles contained more than their usual proportion. The substance of the brain, when divided, was full of bloody points, the veins of the plexus choroides were distended, but all the arteries were empty.

It is worthy of observation in this case that all the blood, which was contained within the head, was in a state of fluidity, as well in the large as the small vessels. Such is not always the condition of this fluid in cases of hanging, and it might have been satisfactory to have ascertained whether it presented the same appearance in other organs of the body. It is well known that suspended respiration, more especially if slowly produced and long continued, will so far affect the natural qualities of the blood, as to render the contents both of arteries and veins one homogeneous mass; but it is far from being certain that such a sudden and short suspension of the oxygenating process, as occurs in submersion or hanging, can destroy the coagulability of this fluid—indeed we know by experiment that it generally does not.

The next five cases illustrate the effects of suffocation from the fumes of charcoal. Four of these patients were sailors, and had retired into the forecastle of their vessel to sleep. On the morning following two of them were found lying on the floor, and two in their beds; but all were equally in a state of the most complete insensibility. The upper part of the chimney it was found had been removed, the fumes of the burning coals had been pent up during the night in the forecastle; thus had the air, in which these men lay, been rendered so sulphureous and impure as to be no longer capable of sustaining life, and when they were brought upon deck no other sign of animation, than slow and labored breathing, was discoverable. *Fairfoot* recovered, however, by being merely exposed to pure air and by the use of a little brandy. *Harman* required at first ammonia and general friction with warm vinegar, and afterwards the removal of some blood from the arm; but *Garbett* and *Jackson* died. In addition to the means employed in the cases of Fairfoot and Harman, oxygen gas was repeatedly inhaled, which never failed to produce some temporary excitement; but respiration became shorter and quicker, and torpor soon returned. In Garbett's case the body appeared of a purplish colour; a considerable quantity of blood flowed from the vessels of the dura mater, which were ruptured by the removal of the calvarium; some serum was effused beneath the arachnoid; the sinuses were gorged with fluid blood; the brain was natural; the lower and back part of the lungs as well as the right side of the heart were also much engorged. The membranes of Jackson's brain were in precisely the same condition; but the brain itself was more vascular, the cineritious substance was unusually dark, there was a small ecchymosed spot in the outer side of the anterior hemisphere, which was limited to the cortical tissue and was unattended with laceration. The sinuses, vena magna Galeni and veins of the ventricles were filled with dark-colored blood. The mucous lining of the bronchi was beautifully injected, the vasa propria of the heart were in the same state, and the right cavities were gorged.

In the 109th case, diseased heart was the cause of the cerebral oppression. A man, aged 30, had complained, about fourteen months before admission into Guy's Hospital, of a sense of weight at the pit of the stomach, followed by shortness of breathing and palpitation. His lips and cheeks were purple, his legs œdematous, the heart beat irregularly and gave to the hand, when placed over the apex, a "sawing feel;" from which the mitral and semilunar valves were supposed to be diseased. At first antimonial ointment was rubbed upon the chest, afterwards a seton was inserted, gentle purgatives and diuretics were administered, and towards the close of life ammonia and other stimulants were required. The right leg was in a state of superficial sphacelus; the right lung was somewhat condensed; the right pleural sac contained some fluid; the left lung also was too solid, and contained little air, except at its edges; the heart was very large; both ventricles were distended and thickened; the mitral valves were ossified to a great extent, the semilunar valves of the aorta were thickened to, probably, six times their natural structure, and adhered by the edges to each other; the tricuspid valves were also a little thickened, the pulmonary artery was large and the aorta small. Several pints of fluid occupied the peritoneal sac. The arachnoid was slightly elevated by some limpid fluid, there was rather too much water in the ventricles, the vessels of the brain wore a dusky purple hue, and the cortical substance a remarkable ash-grey color.

The 110th, 111th, and 112th cases contain nothing of particular interest; but the six succeeding histories well illustrate the connexion existing between diseased kidneys and cerebral effusion.

(113.) A woman, aged 40, labouring under general dropsy, came under the Doctor's care on the 10th of Dec. 1829. The liver was suspected to have been in a state of disease, but from the quantity of water accumulated in the abdomen, the most minute examination of the right hypochondre was unsatisfactory. More than six months previously she had been cured in the London Hospital, by means of mercury, of a somewhat similar attack; in August she was confined, and in about a month after her confinement the disease re-appeared. She was much swollen, the countenance was rather livid, and she was unable to lie down in bed. Her urine was very scanty, of a dark colour and coagulable by heat. Her stools were of a pitchy blackness, evidently from their admixture with blood. A pill, composed of squills and mercury, with nitrous æther, camphorated mixture and liq. acet. am. were at first tried; but on the 26th elaterium was substituted, in doses of a quarter of a grain at bedtime, with five grains of Dover's powder. On the 2d of January she was much better; on the 5th the pills did not act so well; on the 12th the flow of urine again increased; on the 24th a sedative enema and fomentations to the abdomen were found necessary to quiet the action of the bowels and to prevent griping; on the 25th the intestinal irritation was relieved; but on the 26th it returned and the stools were not only watery and purged, but mixed with blood; and on the 6th of February she sunk, without any obvious change of symptoms, with the exception of considerable drowsiness. There was some fluid in the chest. The right lung presented a few adhesions, the bronchial tubes were darkened and filled with mucus, but the pulmonary tissue was healthy. The superior lobe was quite consolidated and filled with suppurating points like tubercles, some of which were empty. The pericardium contained three or four ounces of fluid. The liver was hard and yellow. The mucous coat of the stomach was red, softened and easily separated. From the valve of the cæcum to the anus the lining of the intestines was covered with unhealthy, sloughing ulcers. The fatty tunics of the kidneys adhered firmly, the kidneys were lobulated, hard internally, and their external surface, which was rough, was thickly sprinkled with white, flaky deposits. "The arachnoid afforded a very good specimen of the pure serous effusion."

(114.) A woman, aged 30, who had been afflicted for four or five years with dyspnœa and occasional spitting of blood, was seized with dropsical symptoms four months before her admission into Guy's Hospital. Her face was full, lips somewhat livid, right orbit puffy, pulse 120, feeble. She lay on the right side, and when she turned to the left, the dyspnœa increased, the countenance grew purple, the veins of the neck were distended, and the right carotid pulsated violently. The impulse of the heart did not seem to be unusual in strength; but its beat was attended by a "rasping sound," which was audible on both sides of the thorax. On the whole the chest was less resonant than it ought to have been. The bowels were open; the urine was scanty and coagulable. No medicines seemed to afford her any relief. Six days after she was admitted her nausea and sickness were distressing, her urinary secretion was nearly suspended, drowsiness supervened, the muscles were constantly convulsed on the eighth, and on the morning of the ninth she expired. Considerable œdema of the lower, but little of the upper extremities. The veins of the dura mater were enlarged, those at the base were also turgid, and a complete layer of transparent fluid was covered by the arachnoid. The substance of the brain was unaffected by disease. There were strong adhesions between the pleuræ of the (right?) side, and the (right?) lung had rather a solid, or tough feel. The heart and pericardium were connected by close and general adhesions, and the former was much enlarged. The right auricle was dilated, the musculi pectinati were much developed, and contained between them small fibrinous coagula. The tricuspid valve was much thickened and contracted, so that the orifice would not admit more than the points of two fingers. The right was as large as the left ventricle, and its walls were greatly thickened. The mitral valves were also thickened and contracted, and the semilunar valves of the aorta were studded with several vegetations of such recent growth as to be easily detached. One of the vertebral arteries arose from the aorta. The omentum formed a band across the abdomen, by having been drawn together by a very slight adventitious membrane, which might be traced over the whole intestines and mesentery, rendering them opaque. The liver adhered to the bosom of the diaphragm, was hard and contained much blood. The lining of the stomach was thick and granular; that of the intestines was œdematous. The kidneys, especially the right, were mottled and granular, and their investing tunic could not be removed without tearing with it some of their substance.

The Doctor is disposed to believe that the first link, in the complicated chain of morbid appearances which this case presented after death, was the connexion between the heart and pericardium—that this unnatural union disturbed the harmony of the heart's action, and tended to disease its valves, and that, with the exception of the very recent vegetations which appeared upon the surface of these valves, the cerebral affection was the last developed, and arose from the congestion occasioned by the disease in the kidneys. What precise relationship exists in such cases between diseased kidneys and affections of the heart it is not easy to describe. These organs are not seldom simultaneously affected, but the Doctor inclines to the belief that a mottled condition of the kidneys has no etiological connexion with disease in the central organ of the circulation.

(115.) A man, who had for no less a period than 27 years been expectorating purulent matter, which was supposed to come through the diaphragm from an abscess in the liver, became at length anasarcous, grew drowsy and desponding. Twelve hours before his death he was seized with apoplexy, blood was taken from his arm, and he seemed for a time to recover; but his stupor returned, and he died completely apoplectic. Some œdema of the face and legs. The lungs were perfectly healthy, with the exception of one small portion of the right lung, which was hepatized. No trace of abscess, nor adhesions to the diaphragm could be discovered. The coronary arteries and patches of the aorta were cartilaginous. The liver was granulated and broken down easily, but presented no scar of former disease. The gall-bladder was full of calculi; the spleen had many cartilaginous deposits upon its surface. Both kidneys were granulated and firmly adherent to their tunica adiposa. About eight ounces of urine in bladder, of a deep color, and throwing down a darkish deposit when exposed to heat. A large layer of serum was contained beneath the arachnoid, which in some spots was opaque. The plexus choroides were diseased, but the cerebral substance was quite healthy.

It had been believed that the matter, expectorated in this case, belonged to an abscess in the liver, which had burst through the diaphragm into the right lung; but neither lung nor liver betrayed any such disease upon examination. The purulent matter, therefore, which was so long expectorated, must have been secreted by the bronchial tubes, and it is to be regretted that the condition of these organs is not alluded to in the post-mortem detail. The chronic anasarca of this man evidently depended on the diseased condition of his kidneys; but it is less certain how far the tumors in the choroid plexus co-operated with this morbid state, in giving rise to the apoplectic attack. Such tubercular bodies have been frequently observed in cases, in which derangements of the circulation have occasioned apoplexy, but whether they should be regarded in the light of effect or cause the Doctor does not decide.

"Of the connection, however, (he observes,) of this morbid alteration of structure, with serious derangement of the cerebral circulation, either as cause or effect, I am strongly convinced from the cases I have seen. In page 97 of the former volume of this work will be found the case of William Wright, who died in a state of complete coma, into which he had been falling for many days; and the only morbid appearance discernible in the brain was a disease of this nature in the choroid plexus. In the Museum of Guy's Hospital, No. 1587, is a tumor of this character, taken from the head of a man who died from sanguineous effusion between the arachnoid of the dura mater and that of the brain. And during the last winter, in a case which came under judicial consideration, and where an individual was nearly suffering punishment for murder, it was ascertained by dissection that death had been caused by apoplexy, with very slight effusion; and tumors of this kind, of the size of marbles, were detected in the choroid plexus; and it is somewhat remarkable that in the case I have just mentioned as preserved at Guy's,—a person was actually tried on suspicion of murder, but it was satisfactorily proved that the death was from natural causes. In the Museum of the late Mr. Heaviside was a well-marked preparation of this disease, in the choroid plexus of both ventricles, where death is said to have been caused by a blow on the head. I know nothing of the history of this particular case, but I am sufficiently convinced of the connection of this disease with fatal cerebral changes to inculcate caution in deciding on the cause of death where these tumors are found to exist." 242.

(116.) A stout, short-necked man, who was suffering from necrosis and had been in the habit of using laudanum to relieve his sufferings, was found one morning in an apoplectic state from which he never recovered. On inspection the arachnoid was found somewhat opake, and enclosing a considerable layer of serum. The substance of the brain was not remarkable for vascularity, the vessels on the lining membrane of the ventricles were turgid, and these cavities contained a decided excess of fluid.* The lungs adhered to the ribs, but in other respects were healthy. The stomach was tympanitic. The lining of the stomach and duodenum was pale and soft. The liver was rather large and full of blood. The kidneys were pale, granulated and almost tubercular on their surface. The urine was very coagulable.

The immediate cause of a death so sudden as that, which occurred in this instance, is not very perspicuously revealed by the autopsy. There was no sanguineous effusion, no extraordinary vascular turgescence, nor a very unusual quantity of serum either beneath the membranes, or within the brain. The man had been addicted to an habitual use of laudanum; it was during night that his apoplexy came on; a bottle, in which that drug had been contained, was found above his bed on the morning of his decease; and the torpor, into which he at first fell, never intermitted for a moment until the close of life. These circumstances are, at least, suspicious that his death may have been promoted by an over-dose of opium. The state of the kidneys, no doubt, contributed to produce the slight serous effusion which existed within the head; and, as the Dr. very justly remarks—it is difficult to consider the state of the choroid plexus as wholly unconnected with the fatal event.

The pathology of the next history is more fully developed.

(117.) A man, who had symptoms of stricture and diseased prostate, and who laboured under great difficulty in passing water three days before his death, became exceedingly drowsy; sleeping day and night, and often so soundly as not to be roused. On the evening before he died he was so overcome by sleep, as to go to bed without undressing. The lungs were rather emphysematous, adhered towards the apex, and contained several hard lumps of carbonaceous and tubercular matter. The mitral valve was ossified, the tricuspid valves were much thickened, and the left auricle was as much distended as the left ventricle was contracted. The pancreatic duct was filled with a grey mucous substance like starch, and its lining membrane was obviously thickened. The spleen was small and covered with small cartilaginous deposits. The kidneys yellow, granulated and very firmly adherent to the adipose tunics, which from their hard surface seemed to have been subject to frequent inflammation. Pelves of kidneys and ureters dilated, bladder thickened, urine very coagulable, prostate enlarged. The dura mater appeared thick and opake, and a layer of fluid "not less than a quarter of an inch deep" was deposited beneath the arachnoid.

In the 120th case unhealthy liver, extensive ulceration of the intestinal

* The large vein of the plexus choroides was full of blood, the plexus itself was of a light drab color and a granulated appearance, and on the right side this became even more marked, as it was followed into the posterior cornu to which it firmly adhered. One part of the left plexus was converted into a mere fleshy lump.

lining, numerous vomicæ in the left lung, and opacity of the arachnoid, with considerable serous effusion beneath it, proved the existence of very serious disease throughout the three great cavities; but although the hepatic affection was the most prominent during life, and the thoracic symptoms must have materially reduced the powers of the system, it is probable that the cerebral derangement was the more immediate cause of dissolution. The irregular habits of the patient had, unquestionably, been long laying the groundwork of disease both in the brain and liver. That milkiness of the arachnoid, which was here observed, is often the consequence of acute inflammation, but more frequently of a slow and tedious process of excitement; and in no instance within our recollection have we found it wholly absent, where a systematic and prolonged habit of intemperance had been indulged in. The next case is very interesting on more than one account.

(121.) A carpenter, aged 45, before his admission into Guy's Hospital, had been ill for two years with anasarca of the lower extremities, for which cupping over the region of the liver, infusion of juniper, supertartrate of potash, and other remedies were employed. About a month after he came under the Doctor's care, he was seized with hemiplegy of the left side, under which he more or less suffered until his death. On removing the skull-cap and dura mater, the brain appeared very exsanguine, and on the left hemisphere, close to the temple, one of the convolutions was excavated to the "extent and depth of half a hazel-nut." This cavity looked clean, and had no resemblance to a recent ulcer. Although a considerable quantity escaped during the dissection, seven ounces and a half of perfectly limpid fluid were taken out of the lateral ventricles. The right ventricle was decidedly the most distended. The heart was much enlarged, and universally adherent to the pericardium. The liver was indurated, mottled and covered with an adventitious membrane, by which the gall-bladder was bound down.

"In this case (says the author) it is not improbable that the condition of the heart laid the first foundation for disease; and when the exposures incident to the life of a labouring man induced irregularity in the circulation, through the other important viscera, the general anasarca and the effusion into the ventricles followed as consequences. There is much reason to suppose that the hemiplegia depended upon the pressure of fluid in the ventricles, and that this was chiefly poured out in the months of July and August, about five months before death: the fact that the left side suffered most, while the right ventricle had been most distended, favours the conjecture; and on the other hand, the excavation in the surface being on the side which suffered most from paralysis, leads us to suppose that it had less influence in producing those symptoms; at the same time it is not quite evident to what cause we are to ascribe excavations of this kind; not improbably they are the results of laceration and injury of the brain, followed by absorption of the injured part." 252.

The seat of disease in *Diabetes* having usually, as it were by some prescriptive rule which has been more easily followed than explained, been referred to the abdomen, few have troubled themselves with examining in such cases the condition of the cerebral organs. Yet it must be admitted that our post-mortem examinations have hitherto found but little abdominal disease to account for an affection so peculiar. It was at first declared by Mead that the liver was seldom, if ever healthy, and Home and Rollo have pointed to the kidney as the certain source of evil; but it is not to be denied that both of these viscera frequently exhibit the most unequivocal marks of health, in cases where this disease has destroyed life. Dr. Bright has generally found the liver remarkably healthy; and, with the exception of their being either vascular or flabby, the kidneys have appeared to him seldom diseased. Often, but not always he has found the lining of the stomach and bowels either inflamed, contracted or ulcerated; and in all the cases, in which the head has been examined, "decided morbid appearances have been found." Serous effusion, great vascular turgescence, an apparent reduction in the natural size of the brain, and a state of structural consistence less firm than ordinary, and yet insufficient to entitle it to the character of "softening"—have been the results of his dissections. These, however, have been too few to warrant any inference stronger than a suspicion that the deranged function of the kidneys in diabetes may have more immediate alliance to some unhealthy cerebral state than has generally been imagined. We know that mental excitement or depression operates remarkably on the urinary secretion; that injuries of the spinal cord often wholly change its character; and that the irritation, produced by an hysterical paroxysm, acts as a powerful diuretic. These are facts well established, which it may be wise to keep in view while this point is under investigation; and this chain of relationship between the brain and kidneys is composed of a double series of links, for when the secretion of the kidneys is seriously deranged we have already seen how strongly the functions of the sensorium sympathize. In ischuria renalis, when urine ceases to be elaborated, the patient sinks under apoplectic symptoms. With his characteristic caution the Doctor refrains from going farther, than to suggest the connexion to which we have now alluded, but gives the three succeeding cases as part of the evidence, upon which the probability of such connexion rests.

(122.) A young man, aged 19, afflicted with diabetes, was placed under the author's care on the 25th of October, 1828. All his symptoms were well marked and severe. The quantity of urine passed during 24 hours was 14 pints, each pint yielding nearly two ounces of extractive matter, which was sweet and granulated like honey when kept for some time. The uva ursi, conium, alum-whey, carbonate of magnesia, sulphate of zinc, fomentations to the bowels, and the warm bath were all tried; vegetable food was abstained from, and he was twice bled from the arm. Of these the magnesia was the only medicine which produced any obviously good effect. Opium, at first in one and afterwards two grain doses thrice daily, was employed with decided benefit. The urine diminished to half its quantity, and the extractive matter was less saccharine. Towards the end of January inflammation of the right lung supervened, which required a blister, repeated bleeding both by the lancet and cupping, and the free use of calomel, opium and tartar-emetic in the form of pill. By the 4th of July he was in all respects better and his pectoral affection had nearly subsided; but on the 10th of the same month the left lung became similarly affected with the right, and the same remedies were employed; but during the night of the 13th he died quite exhausted. The body was much emaciated, the integuments were almost like thick paper. The heart was small. The mucous membrane of the cæcum and colon was eroded in some parts, and in others in a state of slough. The kidneys were rather large, firm and of a deep chocolate color.

The arachnoid contained a large quantity of serum, the vessels were distended in a remarkable degree. The ventricles were also distended; the brain was flaccid, and the cerebellum was so very much so as to amount almost to a state of softening. The left lung adhered to its lower and posterior part and a most marked and curious appearance presented itself.

"A portion of the pleura costalis of nearly an oval form near the angles of the ribs was inflamed, in length about six inches, and three inches broad at its widest part. On this, when the lung was gently torn away, was left a thick layer of coagulable matter, at the lower part opake and yellow, but the greater part looking like a yellow gelatinous semitransparent deposit; this was composed of fine filaments of fibrinous matter, containing a yellow fluid in its meshes. The membrane on which this lay, for the extent of an inch all round, was almost purple with vascularity, which, on close examination, assumed the most beautiful dendritic forms. When this portion of pleura was stript from the ribs, the lower side was covered with bloody points, on which stood drops of blood, showing how completely in a thin subject such as this the vessels of the pleura are under the command of local bleeding by cupping or leeches. The corresponding portions of the pleura pulmonalis were nearly in the same condition.

Besides this state of the pleura, the lower lobe of the lung itself had been inflamed almost through its whole extent, and was in a state approaching to hepatization, but combined with some œdema, and on the lower part of the lobe was a circular patch of gangrene of the size of a crown-piece, which showed the limits of a gangrenous mass beneath, proceeding towards the part lodged on the diaphragm, where two circular patches of the same character were seen. This lower surface of the lung adhered to the diaphragm by thick layers of lymph, as the other part of the lung did to the pleura; and the diaphragm all around was inflamed, so that the deep purple inflammation pervaded to the lower surface of that muscle; and even the spleen, which here came in contact with the diaphragm, was rendered of a dark colour at the point of contact. The gangrene of the lung at the part was complete, forming a green mass, more like mud than pus, and smelling most offensively: it had not apparently discharged itself at all, but was pretty well separated by a thin membranous division from the neighbouring part of the lung. In the same globe, higher up and at the distance of two inches, was a circumscribed cavity of the size of a hazel nut, full of the most perfect yellow pus. The upper lobe was nearly natural, a little emphysematous, and containing some serum. No tubercular deposit had taken place.

On the right side of the chest precisely the same process had been going forward, but the disease of the pleura was not quite so extensive, whereas the disease of the lung was still more so, and both the one and the other were in the more advanced stage. The adhesion to the pleura was much more firm, so that the gangrenous portion of the lung could not be separated without lacerating the pleura of the lung, and thus opening into the cavity, which appeared to be making its way through the skin between the ribs, though it probably would never have done this, as it was found to communicate very freely with some large branches of the bronchi by which it had evacuated much of its contents. In the centre of this large gangrenous cavity was a slough, still connected loosely to the sides, and hanging from a kind of stalk, where the lower surface of the lung lay upon the diaphragm. On this side the adhesion was more firm than on the other, and numerous straight vessels were seen running from the one surface to the other in the fine filaments of the newly formed membrane. The middle lobe was hepatized in some parts; the deposit in its structure assumed somewhat the aspect of tubercular matter deposited in lumps, but no complete tubercles were found. The upper lobe was like the upper lobe on the other side." 258.

(123.) A man, aged 26, was seized with symptoms of diabetes mellitus in a severe form. He was much emaciated, his skin was dry, eyes glassy, gums tender, bowels costive, appetite voracious, thirst constant and urine passed in the quantity of six pints during the 24 hours. Various remedies and systems of cure were tried, amongst which animal diet and hydrosulphuret of ammonia were the most rigidly persisted in. Free purging at one time appeared to do good, and the urine diminished under the use of opium, of which he took from one to three grains three times a day for some weeks. Towards the close of the disease one of the most prominent symptoms was a growing imbecility of mind, attended by a dimness of sight. On inspection both lungs were found to contain in their upper lobes large cavities, lined with cartilaginous membranes, and filled with pus, besides many tubercles. The heart was small and the cellular membrane investing it, more particularly at its apex, was dropsical. Liver extremely healthy, and kidneys perfectly so both in color and consistence. The stomach and intestines bore no traces of disease. Between the arachnoid and brain a considerable quantity of serum was deposited. The ventricles contained at least six ounces of limpid serum. The septum lucidum was so attenuated as to allow the fluid to pass freely from one ventricle to the other. The smaller ventricles were also filled with serum. The substance of both brain and cerebellum was soft and watery.

(124.) "The following case of diabetes presents, in the morbid appearance of the brain a striking similarity to the other two I have detailed. I saw but little of this patient during life though I observed his emaciated appearance and examined his diseased urine occasionally, as I passed through the ward." The body was much emaciated. The liver was very healthy. Kidneys firmer than usual, if in any respect differing from their ordinary character. Other abdominal and all the thoracic viscera healthy. The arachnoid was quite distended with fluid; the veins were turgid, cerebral substance flabby, but not softened and very much gorged with blood; the ventricles contained more than their usual quantity of fluid, and some lay at the base of the skull.

There are few diseases more boldly featured than *Apolexy;* few whose symptoms are generally esteemed less equivocal, yet in many of the cases which follow the truth of the observations, with which this article opened respecting the equivocality of symptoms, is abundantly confirmed. According to the Doctor's experience the people attacked are neither uniformly tall nor short, spare nor corpulent, young, old, nor middle-aged. In some there was no premonition, in others it was most marked; in some the attack occurred without pain, in others it was severe; sometimes consciousness was merely impaired, at others it was wholly lost; in some cases palsy followed, in other instances no vestige of disease remained; at one time the nerves of sensation were alone affected, at another those of motion, and in a third palsy had seized equally alike on both; in some convulsions are frequent, general and severe, while in others they are wholly absent; sometimes hemiplegia, at others paraplegia supervenes; in some cases great pain is experienced in the palsied part, in others none; in some the mind is weakened, if not irreparably shattered, while in others the mental injury is so slight that it is only by close examination it can be detected. What relation such varieties and changefulness of symptom have with the situation or extent of the effused fluid is a question of very considerable interest, but difficult of solution. The almost universally admitted fact, that palsy occurs on the side opposite to the seat of pressure is, as we shall see, questioned by the 145th case, in which the left side was the subject of both the hemiplegic and cerebral disorder. The Doctor has found that great pain was often complained of when the fluid effused was situated upon the surface of the brain —that the most rapidly fatal cases were those in which the fluid had been effused into the ventricles—that there was often some connexion between some injury of the cineritious substance, or ventricular effusion, and convulsions—that the corpus striatum has been found injured when deglutition and speech were seriously impaired—that, in accordance with Serres and Foville's opinion, injuries of the corpus striatum and anterior part of the brain are followed by palsy of the lower extremities, while those of the optic thalamus or posterior parts are succeeded by palsy of the upper extremity of the opposite side—that when blood is effused externally to the brain it may be found either between the dura mater and arachnoid, beneath the arachnoid alone, or in both situations at the same time—that the choroid plexus may be ruptured, and thus give rise to apoplexy; and, finally, that the most frequent site of effusion is a little to the outside of the corpus striatum in either hemisphere, where the arterial trunks give off large vessels in the fossa sylvii. These are the leading general results to which the Doctor has been brought by his experience in apoplexy, and many of them are as curious as some are important. But he modestly observes that "the deductions, which I have been able to derive from my own observation, are few and not sufficiently confirmed, to be advanced in any other way than as hints for inquiry, or as confirmations of the observations of others, when they agree." 329.

The Doctor has brought forward no fewer than 42 cases of well-marked apoplexy, and as no two of them are in all respects alike, the reader will find in their attentive perusal as much experimental knowledge, as falls to the lot of even our most fortunately circumstanced practitioners. It would be inconsistent with our limits, however, to lay before him more than a few of the most interesting—the rest ought to be consulted in the pages of the original.

(125.) A young man was suddenly seized with insensibility and stertorous breathing, for which Mr. Streeter's assistance was required. As the pulse felt jerking and the temperature of the skin was considerable, a vein was opened, but scarcely had two ounces issued from a small orifice when he became cold and the pulse sunk. Cloths dipped in hot water were applied to the epigastre, and stimulants were given internally ; but as the head felt hot and the carotids throbbed violently, cold cloths were preferred for the temples and forehead. An attempt was made to draw blood from the nape by cupping, but the pulse fell so much after the abstraction of an ounce, that it was not considered prudent to persevere. Purgative medicine was given with some relief, he regained his speech, and in part his sensibility, but the fit repeatedly returned, and in little more than a week he died. About eight ounces of blood were effused over the left hemisphere of the brain, which had proceeded from the bursting of a small aneurismal sac situated at the union of the anterior and middle lobes. This sac contained a clot, which had nearly closed up the aperture, and although its connexions were destroyed before its nature was discovered, it appeared to have belonged to one of the branches of the middle cerebral artery. There was rather too much water in the ventricles.

(288.) A married man, aged 45, who had experienced several slight attacks of palsy, complained of headache, staggered and would have fallen had he not been caught. He attempted to speak, was somewhat convulsed, soon became senseless and never after spoke, or swallowed. His left pupil was dilated, his right contracted, he breathed stertorously and lay in a state like tranquil sleep. He was freely bled and had some croton oil smeared upon his tongue; but he expired thirteen hours after the first attack. The vessels of the dura mater were turgid, especially on the right side, the convolutions in both hemispheres were flattened, a layer of coagulated blood was distributed in the cells of the pia mater pretty generally over the left hemisphere; the cerebral substance was healthy; the two lateral ventricles were distended with "fluid serous blood;" the inferior connexion of the septum lucidum was soft, ecchymosed and torn. The substance of the left corpus striatum and of all the three lobes of that hemisphere was occupied by one extensive cavity containing blood. The coats of the right cerebral artery seemed healthy, but the cylinder was filled with a firm pink-colored cord of fibrin, which ramified into one or two of its branches. On blowing into the left trunk, as it entered the fissure of Sylvius, very minute ramifications were inflated; but it could not be ascertained whether the aneurismal sac were filled. A secondary arterial ramification showed a small aneurismal dilatation, filled with dark solid blood.

(126.) A lady suffered an attack of apoplexy, accompanied with severe pain in the top of the head, which descended down the back and spine, and which was relieved by bleeding. Nine months afterwards she was again attacked and bled to a few ounces, but died after having experienced a third fit. Dissection could discover no vestige of the former attack; but the surface of the brain, particularly on the left side, was covered with blood, and there was some in the third ventricle. The choroid plexus was pale, and it was believed that the rupture had taken place at the union of the vena magna Galeni with the lateral sinus. In the next two cases apoplexy was induced by a fall, and blood was effused upon the surface of the brain. The 129th case is somewhat peculiar for the remarkable character of the circulation, the pulse having ranged between 22 and 30 beats in the minute; and for the excessive hypertrophy of the heart, having been at least twice the natural size.

(130.) An elderly gentleman, of temperate habits but of an anxious mind, retired to rest much fatigued after the labors of the day, after having moderately supped. About half-past one he awoke with feelings of nausea, and after vomiting he was relieved. His wife became alarmed by some noise which proceeded from his throat, and when assistance came he was found in an apoplectic state, which destroyed him in twenty minutes. The membranes of the head were turgid with dark-colored blood; the substance of the hemispheres appeared generally healthy; the lateral ventricles were distended with semi-fluid blood, which had torn through the commissurà mollis, and entered through the third into the fourth ventricle. The surface of the corpora striata was broken down to about the depth of the fifth of an inch, and the velum interpositum was rent. The source of the blood was not discovered; but was suspected to originate in the lacerated portion of the septum.

How far the vomiting in this instance contributed to the hæmorrhage may be a question, and it may indeed be doubted whether in any degree it conduced to that effect. As the Doctor justly observes, there is good reason for believing that the nausea arose from the cerebral disturbance, which preceded the attack; but there is less for ascribing the attack itself to the vomiting which followed it.

(135.) A stout built sailor, aged 61, fell from some part of the rigging of his vessel and was somewhat injured. He was bled to a pint, after which he was brought to the hospital, complaining of headache and palsied on the left side. A pint and a half more blood was drawn and a purging pill was given with considerable relief to the pain in his head, and shortly after 12 ounces were removed from the nape by cupping, cold applied and a purging enema ordered. Two days afterwards he had a severe fit resembling epilepsy, which continued two hours, and left him nearly senseless, and during the next day he died. Heart large; arch of aorta thickened, uneven in its entire course and, before dividing into the iliacs, its inner surface was broken and in one point ulcerated. The carotid, vertebral and basilar arteries also diseased. The convolutions of the brain were considerably flattened, and on cutting through them a softened portion was discovered in the centre of the middle lobe of the cerebrum on the right side, forming the top of an apoplectic clot, which was situated in the middle lobe of the right side, and contained about an ounce of blood. The right ventricle was not penetrated; but its lining membrane was the only partition between it and this effused fluid. The kidneys were granulated. The urine albuminous.

(143.) A man, aged 47, who had been Clown to one of the Minor Theatres, and was said to have been subject to some head-affection for two years, was admitted into hospital in a state of great depression, with fluttering pulse and cold extremities. A little wine and some subcarbonate of ammonia were given to bring him out of this state of collapse, and next day he was delirious, restless and talked unintelligibly. His head was shaved and covered with cold, a blister was placed between the shoulders, and a pill, composed of one grain of calomel and four of hyosciamus-extract, was given every six hours. The next night was passed more tranquilly, but his speech became inarticulate. Three days after pectoral symptoms appeared, yet his pulse was 80 and weak; during the next two days he grew worse, and on the ninth day after his admission he died in a state of great feverishness, with a hot skin, purple cheeks, dry tongue, and a pulse at 120. Portions of the arachnoid were opaque and some fluid lay beneath it; the carotids, basilar, and arteries passing over the corpus callosum were much ossified. The cortical brain was soft, and when pinched between the fingers "a thin layer, about one third or one half of the thickness of the whole separated quite smoothly from the rest, and allowed of being peeled off in flakes as large as a shilling." The medullary texture was firmer than usual; the ventricles were distended, and their lining was thickened, scabrous, and in some points adherent to its opposite surfaces. On the right side there were vestiges of two or three apoplectic cells; one small cavity was seen in the back part of the centrum ovale, a similar appearance was discoverable in the centre of the left cerebellum, and a large cyst occupied the centre of the corpus striatum in the right side. Each of these cysts was bounded by firm walls, and filled with a yellow transparent fluid. A quantity of turbid serum, mixed with flakes of coagulable lymph, occupied the right chest, and the right lung was condensed; the pericardium contained some gelatinous fluid; the surface of the heart was covered with coagulable lymph, and there was great hypertrophy of the left ventricle. The liver was slightly, the kidneys much granulated; the inner coat of the stomach was rough and hard, and the urine coagulable by heat.

In this case the brain was very extensively and variously diseased; but its pathological products appear of more ancient date than that of the last illness. The apoplectic cells bespeak the past occurrence of various cerebral attacks; the thickening of the ventricular lining and its adhesions to different points of its own surface shew the prior existence of inflammatory disease; and the disorganized condition of the vessels of the brain, viewed in connexion with the hypertrophy of the heart, may in part explain the frequent derangements which took place in the cerebral circulation.

"The very peculiar condition of the cineritious substance must not be lost sight of, difficult as it is to explain the morbid action of which it was the result. It is a matter of frequent observation, that the cineritious substance is divided into layers, as far as colour is concerned; and in cases of close adhesion of the pia mater from inflammation, I have found a complete lamina of the brain tear off, leaving a well-defined surface below. But in this case, without evidence of inflammation, this separation was more remarkable, though there was no adhesion whatsoever of the pia mater; and I shall shortly have occasion to relate a case where the same morbid appearance was strongly marked, and where, as in this case, the vessels were ossified, and the cerebral substance was very firm; where also vertigo, slight paralysis and occasional incoherence of mind were the leading symptoms during life." 304.

(145.) A middle-aged man was admitted into hospital with severe symptoms of bronchitis and œdema of the lower extremities. His left arm and hand were considerably palsied, and he hesitated in speaking. These were the remaining effects of a complete attack of hemiplegia of the left side, which he had suffered about a year previously. After having spoken to the nurse a few minutes before, he suddenly expired on the third day after he had been admitted. The arachnoid and pia were vascular, firmly adhered to the lateral and outer part of the left hemisphere by a thin yellow flake of fibrinous deposit, and contained beneath them a small quantity of serum. Immediately below this adhesion, and extending to the middle of the corpus callosum, was an opake hard portion of brain, which contained a cavity filled with soft curd-like substance, and was as large as the half of a small French bean. The rest of the cerebral mass was quite healthy. The lungs were emphysematous, the larger bronchi were filled with pus, and the mucous lining was very red and inflamed. The right cavities of the heart were enormously gorged with blood. The interesting peculiarity of this case consists in its furnishing the pathologist with an exception to the general, we had almost said universal and indisputable rule, of having the palsied member and injured portion of brain on opposite sides of the body. The medulla oblongata was unfortunately so injured, that it could not be ascertained whether any peculiarity existed in the course and decussation of its fibres. Supported as we are by several unimpeachable authorities, it cannot be denied that cases, similar to the present, have before occurred; but they are not only so few in themselves, but their very existence so much depends upon the accuracy and honesty of the friends of the deceased, as well as upon the testimony of the medical witnesses, that they can never be regarded as affecting the general rule, in any other way than by establishing its general application. *Exceptio probat regulam* is an old maxim, which may be applied to medicine with as much truth as to philology, astronomy or mathematics.

We find that space will not permit us to extract more than two other cases, each of which is highly interesting.

In the first (146), a middle-aged man, of irregular habits, suddenly lost the use of his thumb and the next two fingers of his left hand, without experiencing any giddiness, or headach. Continuing at his work the whole arm became palsied in four days, and shortly after his left leg grew weak and his tongue faltered. When he came under the Doctor's care the palsy of the left arm and leg was complete, his mouth was drawn to the right side, he had some pain of occiput, his bowels were confined, and his pulse was 84, of good strength. Ten ounces of blood were drawn, and a purgative was ordered. He found his pain worse from the bleeding, and his left hand became affected with severe spasmodic shaking. Twelve ounces were taken from his nape by cupping, and the purgative was repeated. During the next three days he felt somewhat better, but on the 9th the headach so increased as to require the cupping-glasses a second time; on the 16th 20 leeches were placed on the temples; on the 18th 20 more; and on the 20th the C. C. withdrew ten ounces of blood from the nape. The relief afforded by each leeching was great, and the sensation of the arm had almost returned; but on the 21st, although the headach remained much relieved, he was very low, inclined to doze, articulated less distinctly, and was decidedly less alert and less sensible than before. On the following day he expired. The examination circumstances prevented for two days, and then the head only was inspected. The vessels of the dura mater were turgid, the convolutions of the right hemisphere were flattened, and when the scalpel was carried into its substance, to the depth of about a quarter of an inch, a cavity was discovered containing above an ounce of serous fluid, at the bottom of which lay a clot of coagulated blood. This cavity was lined by a very vascular membrane, in thickness about one-twelfth of an inch, soft but tough, and so firm as to bear grasping with the forceps. Its vessels were both numerous, large and convoluted, and lay occasionally so superficially as to permit a bristle to pass under them, while passing from one irregularity on the surface to another. There were four projecting nipple-like points in the cyst, which were so deeply colored as to appear stained by extravasated blood.

Besides this cyst there was a smaller one above, behind, and on the inside of it, which contained about half a dram of fluid without a clot, and was lined with parietes, collapsed, corrugated, and of nearly double the thickness of the larger cyst. The ventricles were in no respect implicated, and the rest of the brain was healthy. The Doctor believes that if this case be viewed as one of apoplexy from ruptured vessels, blood was effused at three or four distinct periods; and, although he is not quite satisfied as to the degree of benefit produced by depletion, he is inclined to consider it upon the whole to have been useful. A more questionable point seems to be, how far these cysts were connected with previous fits of apoplexy. It will be perceived that the parietes of these cysts were equally remarkable for their density and their firmness, and it is generally admitted that a very considerable period is required, before Nature can bring her restorative process to a degree of perfection so matured. In some cases the constitution makes no effort to repair the injury, but the brain softens, fresh attacks of hemorrhage occur, and the disease ultimately proves fatal. In others the effused blood and injured portion of brain mingle into one soft mass, and remain for many months unabsorbed. In some, as in this instance, a cyst is formed to contain and separate the diseased part from the rest of the cerebral structure, this matter is gradually absorbed, the cyst corrugates and contracts, and nothing can be ultimately found but a yellowish-white solid line of substance resembling a cicatrix. But in such instances as this the restorative process commences and is completed at very different periods. In the 136th case it had evidently commenced on the sixth day, and in the 137th on the tenth. In the 140th considerable reparation had been made in 23 days, in the 139th a distinct cyst had been formed in the course of seven weeks, and in the 145th case the apoplectic cavity had been almost obliterated and a cicatrix had formed, but we unfortunately possess no data from which we may conjecture the length of time occupied to effect this. In the 138th case no appearance toward healing had manifested itself after the lapse of 12 days, and in the 144th instance the disorganized mass shewed no tendency to undergo a favorable change when twelve months had expired. These facts being considered it becomes a question how far we may regard the two cysts in the case last given as vestiges of apoplectic disease—

"As I have never (says the Doctor) met with a similar morbid appearance, I am led to compare with it any approach which I may have witnessed, and with this view would refer to the condition of the parietes surrounding the sanguineous clots in the cases of MARY AGNES and of JOHN PUGH, in both of which we find vessels beginning to ramify upon the broken surface, and other larger vessels dissected out so as to be prominent, and even in parts detached from the brain, like some of the larger vessels in the present case. We can easily suppose that vessels thus freed from their usual restraint, and brought to the surface, would admit of great distention, and might form something very much approaching to the vascular plexus composing these cysts: still, however, if this be the theory of their formation, the time allowed appears inadequate; and if such be the case, and we have to look to some previous attack of which we have no account, we may as easily suppose that the vascular cysts were morbid structures not originating in apoplexy, but filled with pure serum, till, by the bursting of some of the vessels of which they were composed, blood was poured out, which by overfilling the cavity would produce pressure and all the symptoms which we have seen; and this latter view is borne out by the fact that in the smaller cyst no bloody clot was found, while in the larger the appearance of certain parts of the surface rendered it highly probable that blood had lately been given out by the vessels, and at the same time the clot was more like recently coagulated blood than we should expect to find in a cyst so highly organized. It is, then, upon the whole most probable that this was a case of hemiplegia from the effusion of blood into a vascular cyst already formed, either by a previous effusion of blood or by some other cause: and the pain experienced during the whole disease depended possibly on the membranous nature of the surface on which pressure was made." 312.

(162.) A man, of active habits, a sanguine temperament, and 48 years of age, was attacked with rheumatism by exposure to cold, and labored under it for many years, together with habitual costiveness and derangement of the digestive organs. Fourteen years afterwards he received a slight paralytic stroke, which affected the muscles of his left side. This attack was followed by several minor ones, which were relieved by cupping, blistering, purgatives and a seton in the neck. The hemiplegia was thus gradually converted into paraplegia, and as the disease advanced one function yielded after another, until he eventually sunk. The mind was, however, unaffected until a few hours before death. With the exception of a little fluid between the membranes and slight congestion of the pia mater, the head betrayed no symptoms of disease. A considerable quantity of serum lay between the membranes of the spinal cord, and the arteries of its pia mater were much injected. "Within the upper dorsal vertebræ on the left side, rather more than an inch in length, in the direction of the spinal axis and about half an inch in a transverse direction, was an apoplectic cell, containing the red and broken remains of a coagulum; and lower down in the spinal canal, the internal ligament was to some extent deeply marked by ecchymosis, as if in progress towards the formation of another similar effusion." This effusion was external to the cord, which did not seem either inflamed or disorganized, and the coagulum appeared to be inclosed in an adventitious membrane.

Apoplexy of the spinal cord is rather a rare occurrence, and therefore the foregoing case is the more valuable. It will be observed that the hemiplegia and the hæmorrhagic deposit were both situated on the left side—that the hemiplegic gradually degenerated into a paraplegic affection, and that the intellectual powers survived until the very last. In all these respects this case differs from ordinary apoplexy, and as it had been mistaken by Dr. Stroud, under whose care the patient was, for apoplexy of the right side of the brain, the reader will do well to note these peculiarities. Dr. Stroud theorizes upon the relationship which maintained between the previous rheumatic affection and this disease of the spinal cord; but we can perceive no ground for theory in this matter, much less can we imagine with the Doctor that it had any "*dependence on mental emotions affecting the spinal cord rather than the brain.*" It is somewhat novel to talk of the mind acting more readily upon the spinal marrow than the cerebrum!

It is almost unnecessary to say that the lancet is our *magnum remedium* in the treatment of apoplexy, whatever tissue or constitution it may invade; but, as the author observes, it may be employed either to remove congestion, or to arrest hæmorrhage. When mere vascular plethora is the compressing agent, depletion produces almost instant cure by unloading the brain of its overplus of blood. It has, indeed, been strongly argued by Dr. Kellie of Leith, and it has been fashionable with some subsequent writers to contend that the brain can contain only a certain quantity of blood, through its being relieved from the pressure of the external atmosphere by the osseous case in which it is protected. But these physiologists have never yet succeeded in reconciling the occurrence of apoplexy with this theory. That the brain is elastic and can be compressed it is impossible to deny; that this pressure frequently arises from engorged vessels is equally certain, and that the removal of this engorgement by depletion is followed by the removal of all pressure-symptoms is matter of daily and indisputable experience. These are facts well known and not easily disputed, and it appears to us that they are totally inexplicable on the supposition that the quantity of blood, circulating through the brain, is under all circumstances the same. If atmospheric pressure be withdrawn from the immediate surface of the brain, as is said, and as it no doubt is, this agent is not withdrawn from the remaining surface of the body, and we apprehend that the very circumstance of the brain being encased within an unyielding cover, while the rest of the body remains subject to all kinds and degrees of pressure, will render it physically impossible for the brain to contain at all periods the same amount of circulating fluid. We can easily conceive, because we can daily see, that inordinate exertion, powerful excitement, vascular plethora, hypertrophy of the left ventricle and such other conditions as materially augment the momentum of the circulating mass, may encumber the brain with more than its natural proportion of blood by compressing its elastic tissue, and may even extinguish its functions by carrying this compression to a degree incompatible with their continuance.

When rupture has taken place and blood has been effused, bleeding must of course be a less frequent and less immediate remedy. *Intravasated* fluid has not only to be withdrawn, that the hæmorrhage may be arrested, but that which has been *extravasated* requires to be removed by the absorbents; and we have already shown how tedious always and how frequently imperfect this department of the cure is. The author deprecates small bleedings, as more calculated to weaken the general powers than to curb the local disease; while at the same time he cautions the practitioner from too indiscriminate an application of the lancet. Very large bleedings, more particularly in relaxed habits, favor serous effusion; and it is well known that by small repeated bleedings universal dropsy has been induced.

"These considerations will render us more cautious in the abstraction of blood, though we must be always ready to meet by bleeding any such aggravation of symptoms as bespeaks a recurrence of congestion, or a tendency to fresh hæmorrhage. When once the circulation has been restored to a more healthy condition, there is no good reason for persisting in depleting measures. We have now to support nature in her operations, and patiently to await the issue: the circulation must be preserved in a quiet and tranquil state, till the injury inflicted on the vessels is healed; and the constitutional energy must be sustained, while a process almost analogous to cicatrization takes place on the surface of the cavity; while the mass of brain mingled with blood is absorbed, and possibly while new cerebral matter is formed by a complicated process of absorption and deposit, which may perhaps distantly resemble the operations which pass beneath our eye when accident or disease has destroyed large portions of bony structure." 336.

When the general system has been previously reduced by venesection, or when the plethora appears so partial as to be limited to the vessels of the head, cupping on the nape or temples may be advantageously employed, and blisters to the nape, tartar emetic ointment, or a seton are advised under similar circumstances; but he speaks with hesitation of the use of cold, except in cases attended by febrile symptoms, or by general activity of the vessels in the brain. Its continued employment, where this action is feeble and there is considerable congestion, tends in his opinion to increase rather than diminish the danger; but he admits that when it is suddenly applied, it may remove congestion if cautiously administered, or even check effusion in cases of rupture. " Employed with this view however, he adds, it would be hazardous, as by driving the blood from the external we might very probably throw it with greater impulse upon the internal circulation of the head." Purgatives are always useful and often indispensible, as a deranged condition of the stomach and bowels is not unfrequently the cause, and still more frequently a consequence of apoplexy. The most active medicines of this class are often required to stimulate these organs to a sufficient degree; but we are cautioned from using calomel where deglutition is very imperfect, lest, as happened to the Doctor, it be retained on the tongue, and induce a state of ptyalism by no means free from danger.

"I once saw most serious consequences result from this, for having put five grains of calomel on the tongue and attempted to wash it down with a cathartic draught, the calomel, instead of passing into the stomach, remained, moved about by the tongue, and produced in a few hours a most alarming ptyalism, in which the tongue was forced out of the mouth, and it was necessary to scarify it deeply before it could be returned within the teeth. If however the patient can swallow well, a dose of calomel with extract of colocynth followed by castor oil is a very proper purgative. Cathartic injections may also be used with much advantage. In some cases, where the paralysis has been less complete, where paraplegia has occurred, or where there has been a combination of hysteric irritation with the paralytic affection, purging has sometimes been the chief means of cure." 337.

After the strength and severity of the attack have passed and our attention requires to be directed to the nervous relaxation, torpor and palsy which too often succeed to it, the balsamic preparations and the mineral tonics will be found of great benefit. The sulph. zinci and solut. arsenical. are especially recommended; nux vomica has been much lauded, but of late its active principle, strychnia, has been usually substituted.

"In a case of local paralysis, I have applied this powerful remedy in doses of the eighth, the quarter, or half a grain, to a blistered surface, with the effect of producing spasmodic action through the paralysed muscle, and I have sometimes administered it internally with advantage. But cases of hemiplegia from the rupture of vessels are not those in which this remedy holds out the greatest prospect of success, though with caution it may be employed in the advanced stages of convalescence with safety at least, and sometimes with benefit. Exercise of the affected parts and friction are of use; and by degrees moderate and cheerful occupation of mind, avoiding excess and anxiety, will rather promote than retard recovery." 338.

It has been already stated that renal and cerebral diseases bear some relation to one another, and cases have been adduced in proof of this connexion. Diuretics might therefore be regarded important medicines, and in practice they appear of very considerable value. Upon what cause the constitutional derangement, which diseases the kidneys, depends, or in what precise manner diseased kidneys act in producing cerebral disturbance, it may be very difficult to determine. In many cases the serum of the blood has been found highly impregnated with urea, but in others there is scarcely any, or none: and from two analyses, lately performed by Dr. B. Babington, it would seem that the quantity of this salt discoverable in the serum of the blood, in cases in which albuminous urine has been passed, is extremely variable. But how far the presence of urea, or of any other principle foreign to the blood, may act upon the brain as an excitant to disease is a curious and important question, which well challenges inquiry. Dr. Bright has certainly demonstrated the presence of albumen in the urine to be a very unfavourable state, which, if long continued, is liable to be attended by such serious disorganization of the kidneys as to admit of little more than palliative treatment. Since the appearance of his first volume of Reports, he has witnessed many cases of this nature, and he is still persuaded that general, or if the strength will not permit, local bleeding over the region of the kidneys, free purging, and the infusion of digitalis in dram doses thrice daily, are the best remedies in recent attacks. This general plan must, of course, be varied by the stage of the disease and other circumstances. When the affection appears confirmed, the union of mercury with the digitalis has proved serviceable; when the constitution is delicate, gentle tonics have been given with advantage; when the bowels are irritable, purgatives must be cautiously exhibited; and as the cerebral circulation must always be regarded, in such states of the kidneys, as in a precarious condition, whenever headache is complained of it should not be neglected for a moment.

We have now described several common and most important sources of *cerebral pressure;* but some still merit notice, which are not rendered the less interesting by their being the more rare. The cases, which follow, illustrate the effects of pressure from the presence of *tumours* in the substance, or attached to the membranes of the brain.

(163.) A talented and amiable man, aged 50, had been for many years subject to gout in the extremities and to occasional attacks of sick-headache. Fifteen years before his death an encysted tumour had been removed from his forehead, and five or six years previously he had received a blow on the head, which stunned him for a few seconds, but was followed by no consequences which attracted notice. In the Summer of 1824 he grew thin, failed in strength and spirits, declined his favourite amusements, and occasionally became abstracted even when in the bosom of his friends. During the Autumn of that year this indisposition to exercise increased, he could not be brought down from his bedroom until the afternoon, his nights were passed in watching and his days in anxiety and restlessness. He fancied that his "head was drawn towards his shoulders," yet upon the most minute examination no other symptom of head-affection could be detected. On the following January the Doctor was consulted for the first time. He was then quite able, though somewhat reluctant, to answer such questions as were put to him; would sit on the sofa and read the paper without attending to it; said that he felt a tendency to be drawn to the left side, and he directed his steps with difficulty, so that when he passed across the room he would sometimes rather run than walk. Occasionally he was very drowsy, and complained of pain in his forehead; he grew irritable and almost irrational on some points, and he became more and more helpless and more abstracted every day. In the Summer of 1825 he went for change of air into the country, and although he could still walk a little he was generally wheeled about in a chair. On one occasion of particular drowsiness a blister was applied to the whole scalp, which brought on erysipelas of the face, strangury and violent spasmodic action of the left hand. In the Winter of 1825 he returned to town, after which he scarcely ever left his room, was fed, dressed, undressed and in short nursed like an infant; but still retained his consciousness, and was grateful for the attention which he received. Sometimes he had drowsy fits of 24 or 48 hours' continuance, with an approach to stertor, out of which he could be only roused for a moment, when he lapsed into his former state. The left leg was often spasmodically moved and the left knee drawn up, his mind wandered and fancied a thousand things; sometimes some strabismus was perceptible, as well as slight distortion of the muscles of the face, deglutition was extremely difficult and imperfect, the tongue was loaded in an extraordinary manner with a thick olive-colored coating, and his pulse was quick and weak, but variable. For many months before death the sphincters lost their power, the skin, which had been peculiarly dry and harsh, became soft and perspired freely for several weeks, he would lie motionless almost an entire day, and though the Doctor was not convinced that his left extremities were palsied, they had not the same power as the right. In May a second physician saw him, and recommended cold to the head and free purging, which however evidently weakened him, and in June he was attacked during the night with an epileptic fit of unusual severity, after which he never wholly regained his consciousness, though he survived the fit about thirty-six hours. The vessels of the dura mater were rather turgid and found to adhere to the anterior part of the right hemisphere, especially about an inch from the front close to the longitudinal sinus. The surface of what appeared to be brain at that part was opaque and hard, and when examined proved to be the top of a tumour which extended backwards almost four inches, and sunk into the hemisphere so as nearly to reach the ventricle. This tumour was covered with vessels, was lobulated on its internal surface, weighed nine ounces, consisted of an uniform cheese-like mass of considerable firmness, and was separated from the thin layer of brain, which interposed between it and the ventricle, by a small quantity of serous fluid. The rest of the body was quite healthy.

The history of this interesting case has been narrated with more than our usual minuteness, that the effects of slight but progressive pressure upon the sensorial functions may be traced. The first stage of declension from ordinary health is marked by no one symptom of a formidable nature; yet the mental powers gradually decayed, and the corporeal functions slowly dilapidated, until the tumour had grown to a size and occasioned a degree of pressure which life was unable any longer to endure. There can be little doubt that, had a foreign body of half the weight or size of this tumour been instantaneously introduced into the brain, death would have immediately ensued; but in this case, as in chronic hydrocephalus, the pressure was at first so limited, and during its progress increased so gradually, that health was at first impaired without being seriously deranged, function was slowly invaded without being suddenly arrested, and the resources of the constitution were permitted somewhat to struggle under and resist such tedious encroachment. It is to be regretted that the symptoms shed such little light upon the source from which they sprung, as they might all have been fairly taken to denote the existence of serous apoplexy. The treatment, though varied, was both active and appropriate, but no plan of cure which was adopted appeared to have produced the slightest good. Cupping was invariably followed by increased drowsiness; leeching gave no relief; blisters, setons and tartar-emetic ointment were applied in vain; purging and tonics were equally unsuccessful.

(165.) An elderly gentleman came under the author's care for palsy of the left side. For many years he had been drowsy in the evening, for the last two or three years he had often complained of vertigo, and during the two or three months prior to the Doctor's visit he had suffered much from headache. While dressing one morning, and in the act of gargling his throat, he threw his head back, felt giddy, and in an instant fell into strong convulsions, out of which he arose with the loss of his left side, save the leg which still retained some power. As his pulse was feeble leeches were applied to the temples, an embrocation to the forehead, and he was ordered a mixture composed of the compound infusion of gentian and tincture of aloes. On the next day he was better and the leeches and mixture were repeated; on the day following the sensation of the left hand improved; but during the next week he on the whole lost ground, and by the end of a fortnight he was deprived of all consciousness and, without suffering any fresh attack, expired in convulsions. The dura mater firmly adhered to the calvarium; the pia mater was very vascular; the convolutions over the middle of the right hemisphere were flattened and softened, and on cutting into the middle lobe of this side two hardened masses were found imbedded in the cerebral tissue, which was much softer than natural, and was not well defined around the margin of these tumours. One of these bodies was as large as a nutmeg, the other was the size of a London plum; the smaller was seated almost at the top of the hemisphere, the larger was nearer to the fossa Sylvii; both had the same structure and were of a lilac hue, with the exception of their centres which were occupied by irregular masses of a light straw color and of superior firmness to the more exterior structure. The fornix was very soft; the corpora striata and optic thalami were natural; the left hemisphere, cerebellum and medulla oblongata betrayed no disease; but the arteries at the base of the brain were slightly cartilaginous, and the left vertebral artery was not above one-tenth of the size of the right.

Our author is inclined to suspect that the first nuclei of these tumors were hæmorrhagic deposits, and that the last fatal attack arose from the extensive softening with which they were surrounded. If this be so, and it is probable from the occasional fits of drowsiness and irritation to which this man had been subject for years, it strongly proves how long the brain may endure a certain degree of pressure, and yet carry on its functions with very tolerable aptitude. In consequence of the unimpaired state of the articulation, Dr. B. had anticipated that the posterior portion of the corpus striatum was exempt from disease, and dissection shewed that this prognosis was well founded. The author is disposed to consider the tumours in both of these last cases as corresponding with those albuminous deposits described in Dr. Abercrombie's Work upon the Brain. In the following instance they were decidedly tuberculous.

(167.) A delicate girl, aged 11, had a fit at school for which she had been leeched and blistered on the head, but from the consequences of which she had not entirely recovered when she was attacked with a second fit of the same kind, for which she applied for admission into hospital. The lower extremities were stiff, cold, shrunk and motionless, but sensible to the touch. The upper extremities were slightly affected, and the right arm was subjected to spasmodic tremors. The back was stiff, the dejections were voided unconsciously, the pupils were dilated, the veins of the scalp swollen, the cheeks flushed, and her screaming denoted considerable pain of head. The head was shaved, cold applied to it, a seton was inserted into the nape, and a purging pill was ordered every night. On the fifth day after she remained unimproved, and on the seventh the abdomen swelled, respiration was effected chiefly by the diaphragm, the pulse was 160, the eyes were directed to the left and motionless, the right arm was rigid, inflexible, and bent across the body, the mind was oppressed but perfectly distinct and clear, the countenance was pale and the appetite, which had been great, was lost. During the night coma, insensibility and hiccup came on, her mouth was spasmodically drawn to the right side, the eyes rolled incessantly, the muscles of the extremities quivered and twitched, and on the next day she expired. The pulse had risen to 176 until a little before dissolution, when it flagged during inspiration and "beat three or four hurried strokes during expiration." The head and extremities were very warm to the last, and a miliary eruption covered the entire chest. Two of this patient's family had died of hydrocephalus. Underneath the arachnoid were visible several irregularly-shaped yellow bodies, which felt hard but did not rise above the surface. Some of these were glued, others more firmly attached to the membranes, and when cut into many of them were found extending into the cerebral substance to the depth of half an inch. On raising the right side of the dura mater precisely the same appearance was discovered. Several smaller but more circular tubercles were found at the base of the cerebrum, always, however, seated as the preceding in the cortical substance, and some were detected in the cerebellum, corpus rhomboideum and cineritious texture of the medulla oblongata. A considerable part of the middle lobe of the left side was softer than the rest, but not obviously disorganized. There was no unusual degree of fluid under the arachnoid, and there was less in the ventricles than usual. No tubercles could be discovered in the spinal cord, which was rather soft; but the lungs, omentum, peritoneum, spleen and kidneys were filled with these morbid growths.

As has been already mentioned, the Doctor has hinted at the specific effects of injuries of certain portions of the brain upon certain functions and parts of the body, and in the present instance he is inclined to ascribe some connexion between the convulsions, which appeared during life, and the tubercular state of the cortical tissue of the brain. It will be noticed that there was no effusion of serum upon the surface or within the cavities of this organ, and so far is the source of this symptom rendered less difficult of discovery. Tubercles appear—

"To affect in a peculiar way the serous membranes, and, not unfrequently, is attended with a disposition to an increased accumulation of fluid in the cavities, showing itself by effusion into the abdomen, or producing hydrocephalus: and it would appear that it is not necessary for tubercles to exist in the brain in order to excite the hydrocephalic action, nor is the effusion of fluid in the brain at all a necessary result of the existence of such tubercles.—Thus in the present case, though the tubercles had proceeded to a great extent, and induced the most distressing effects, no effusion had taken place: and in a case which I shall immediately relate, the effusion had taken place in a constitution exactly analogous, as proved by the other morbid appearances, without any tubercular formation in the brain; while in a third, to which I shall also refer, both the tubercles existed to a small extent in the membranes of the brain, and hydrocephalus had taken place;—from which different facts, it would seem that the hydrocephalus is rather the result of the constitutional tendency than of the tubercles, though these morbid deposits are probably often the exciting cause." 361.

It occasionally happens that symptoms of *cerebral pressure* appear in a very aggravated form and even terminate in death, without leaving behind them in the brain any visible tokens of special disease. The membranes appear neither vascular, inflamed, nor thickened; the cerebral substance is apparently unchanged in texture, and there is neither upon its surface nor within its cavities more fluid than should exist in a healthy state. In some such cases the brain appears of increased volume, filling up the calvarium more closely than usual, as though intertextural nutrition had exceeded its wonted limits. This hypertrophied condition of the sensorium produces symptoms of pressure, similar both in their nature and degree to those occasioned by effused lymph. The following is a good illustration of this rare affection.

(171.) A man, of 45 years of age, who had been employed in a white-lead manufacture for six months, was suddenly seized while at work with loss of sense and motion, which was succeeded by stupor and occasional delirium. When admitted into hospital he could be roused with difficulty to answer questions, when he opened his eyes there was manifest strabismus, his bowels were costive, his tongue was loaded, and his pulse was 44 and labouring. The head being shaved cold was applied, fourteen ounces of blood were drawn from the nape, which was afterwards blistered, and a strong purgative was ordered. After passing a restless night ten ounces were again taken from behind the ears, and one grain was prescribed every second hour. He passed another sleepless and restless night, but he was more easily roused, his pulse stood at 76, and he saw distinctly. Sinapisms were applied to the feet, and his calomel was continued. Two days after he was cupped a third time to twelve ounces from the temples, constant violent delirium gave way to profound stupor, and his stools and urine were passed in bed, but without betraying any other paralytic symptom he expired on the following day. The dura mater appeared quite full and closely embraced the brain, which was so flattened upon its surface that the convolutions were marked only by the most superficial lines. As no fluid lay beneath the arachnoid the membranes separated from the brain, in consequence of this tightness, with difficulty. The cerebral substance was firm and natural, but the "medullary part conveyed the idea of being in great abundance and massive." The ventricles appeared small, and each contained only half a dram of serum. The vessels at the base of the brain, as well as those of the cerebellum and medulla oblongata, were healthy; "so that the only disease was the compressed condition of the surface of the brain, occurring in spite of the total and unnatural absence of fluid, or of any other obvious cause which could occasion it." There was no disease of consequence in any other portion of the body.

In 1824 Hufeland published some observations on *cerebral hypertrophy*, and the author has seen a tendency to it in several other instances, particularly in children: but still it is a rare disease, and, like most others of this organ occasioning pressure, it is not marked by any symptoms so pathognomonic as to point out the source of mischief with any certainty. In other cases the brain seems oppositely conditioned, being in an apparently cachectic state, of diminished volume, and of increased consistence, as though condensed by prolonged pressure. In case 172 this state of brain is well exemplified by Dr. Hodgkin.

An elderly woman, whose mental and corporeal powers had been gradually decaying for some years, often felt a dull pain in her head, which was somewhat relieved by pressure, and was attended by giddiness and a drowsy vacant aspect of countenance. When first seen by the Doctor, in addition to these symptoms she had irregular bowels, a remarkably red tongue, and a general bad state of health. Leeches were repeatedly applied, but she experienced more relief from tonics and laxatives, and her appetite became inordinate, when compared with the relaxed condition of her general system. Her strength, notwithstanding, declined, she passed her evacuations in bed, a slough formed on the sacrum, a diarrhœa came on which astringents could not control, and she ultimately sunk. The dura mater adhered with unusual firmness to the scull-cap, and in some parts was rough and scabrous. The internal surface of the scull was also rough. The arachnoid was healthy and it contained beneath it scarcely more moisture than natural. The sulci on the surface of the brain were wide, and—

"The convolutions themselves, instead of appearing to fill the whole space and gently press upon each other, producing a comparatively smooth surface, looked contracted, crisp, and corrugated, falling into slight hollows or dimples on those parts which are usually most prominent, and marked by shallow transverse depressions traversing the tops of the convolutions. The membranes came off easily, and then the corrugated appearance of the convolutions was more distinct, so that in some parts they almost assumed a notched form; and this morbid alteration, which was observable throughout, was particularly marked upon the anterior portion of the middle lobe on both sides; and some of the small projecting portions presented rather a lighter colour than the other parts of the cineritious matter. When a convolution was taken between the finger and thumb, it gave a sensation of unusual resistance; and when pinched, the external layer of the cineritious substance was detached from the rest with great facility." 374.

The cerebral tissue was firm, but the inner part of the cineritious substance was softer than that more externally situated, and the medullary tissue was firmer at the edge than elsewhere. The ventricles were contracted, their parietes resisted the knife, and they contained very little fluid. The surface of the optic thalami was corrugated, the plexus choroides had many vesicles on their posterior part, and the large vessels at the base were marked by a few opaque patches.

Sometimes the brain is neither contracted nor dilated, but very much increased in firmness, so that the cerebral vessels are compressed and the circulation retarded. In other instances again it is soft, flaccid and tenacious, more especially in constitutions broken down by lengthened illness; and occasionally this "softening" which is not very rare, degenerates into a "watery condition" of the whole mass, as in some cases of diabetes and consumption. Difficult, no doubt, it is to account for the origin of disease in such cases, or to explain its *modus operandi* in causing death; but it is certain that a careful dissection can always disclose a state of brain very different from that of health. It happens, unquestionably, that palsy may occur and yet the brain betray no visible marks of derangement; but in all such cases its membranes should be carefully inspected, as experience has shewn that meningeal disease can induce paralysis as certainly and in as severe a form, as most palpable and extensive disorganization of the nervous mass.

"Cases of this kind are often preceded by rheumatic pains, and sometimes by distinct attacks of rheumatic gout, sometimes by well-marked neuralgic affections; and they are frequently to be traced to exposure to cold and wet. In some of them, examination after death gives satisfactory evidence of the existence of such results of inflammation as are well calculated to produce pressure, and at the same time proves the nature of the action which has been going on; but in other cases the appearances are scarcely sufficient to account for the symptoms. Still it is right that we should take into consideration the peculiar structure of the mass of nervous matter which composes the brain, and the numerous and complicated bundles of fibres of which the spine and nerves are formed, and the extent of the fine membranes to which I have just referred. Supposing, then, that the membranous structures are extensively influenced by morbid action, we easily understand how in many cases all the effects of pressure, or at least of interrupted circulation, should be induced, without the brain or nerves presenting any very great or obvious appearance of disorganization.—Nor is this view of the matter imaginary, but it is the conclusion to which I think we are fairly led by contemplating the slight but obvious changes which are very frequently exhibited in cases of a similar kind, and which will be learnt from some of the following cases, presenting themselves in the forms of thickened membranes,—slight adhesions,—thin adventitious deposits,—and serous effusion more or less extensive." 375.

In the 173d case a middle-aged man, a basket-maker by trade, at first experienced a sense of numbness in his legs and thighs, which ascended to the lower part of the body, and at length reached the upper extremities, but was particularly great in the little finger and that next to it of each hand. Setons, blisters, tartar-emetic ointment, moxa, nux vomica, the mineral solution, the shower-bath, Peruvian balsam, and free purging were successively tried in vain; head-ache at last came on, delirium followed, and he expired. The dura mater was remarkably vascular, thick, tough and firmly adhered along the glandulæ pacchionæ and anterior part of the right hemisphere to the arachnoid, which was opaque, much thickened, and contained beneath it a moderate quantity of fluid. The ventricles contained four ounces of limpid serum, but the brain was healthy. The foramen magnum was much diminished by a thickened state of the ligaments. The spinal cord was very small.

In the 175th case the disproportion between the post-mortem appearances, cerebral disease and symptoms which accompanied it during life, is very striking. Suddenly, as it was supposed, from exposure to cold, the patient completely lost her sight, yet the only part of the brain, in which any deviation from a healthy state was discoverable, was in the medullary portion of the optic thalami, which however presented no visible change in structure or consistence, being merely discolored to some extent with an opaque light buff. She was also with equal suddenness deprived of both the power and sensation of her lower extremities, and the bladder had become palsied, so that her urine accumulated on one occasion to seven pints and a half; yet nothing unnatural could be detected in the spinal cord, with the exception that its surface presented a series of transverse elevations "as if it were too firmly bound by the investing membrane," and there was nothing worthy the name of disease in the brain or cerebellum.

In the 177th case general paralytic symptoms came on after exposure to wet and cold, and, although they were combated by bleeding, purging, &c. they proved themselves beyond the influence of medicine. The dura mater was found healthy, but the arachnoid was slightly opaque and covered some fluid. The brain was natural in appearance, but the ventricles contained too much serum. The spine could not be examined.

Absorption of *lead* into the system is a well known and not an unfrequent cause of palsy; but as it has not yet been shewn whether any other portions of the nervous system are affected than those nervous masses, which immediately supply the palsied organs, *paralysis from lead* is generally regarded in the light of a local disease, requiring more especially local treatment. The intestinal tube and upper extremities are the parts, upon which this poison more immediately operates; occasioning constipation of the most intractable form, and palsy of the extensor muscles of the forearms, by which they are more or less deprived of the power of raising the hands. Young plethoric persons, just fresh from the country, are its easiest victims; and the Doctor believes that while the painter is more frequently attacked with palsy of his arms, the lead-manufacturer is more obnoxious to the abdominal affection. Exposure to cold and wet, neglect of personal cleanliness, and the intemperate use of spirituous liquors, evidently predispose the constitution to the operation of this poison. In some cases the very freest contact with this mineral for years is succeeded by no bad effect; while in others it cannot be handled for as many days with impunity. In one instance a waterman lost the power of his hand without any previous ailment, from having painted his boat. The Doctor is somewhat inclined to suspect that however this nervous affection may appear to be local, the brain and upper part of the spinal cord are not always unaffected. As he justly remarks, there are two sets of symptoms in this disease—one of which betoken intestinal disorder, as colicky pains, furred tongue, sickness of stomach, a tympanitic state of the abdomen and constipation—the other a paralytic affection, which is often ushered in by headache, vertigo, and a sense of weight and pain between the shoulders. These latter symptoms are supposed by the Doctor to denote some cerebro-spinal irritation; and although he has not had any opportunity of substantiating this suspicion by examination, it has received very considerable countenance by the relief which has followed cupping from the nape.

(184.) A man, aged 36, who had been painter for 25 years, but had never suffered from colic, seven years before admission into hospital was seized with giddiness, headache and the sudden loss of his hands. When attacked he had been engaged in *flatting*, a process it would appear peculiarly noxious in its effects upon the body; for nine months he required to be fed, but he ultimately got well and recommenced his occupation. Two years afterwards he was again seized by the same symptoms, which again disappeared after continuing four months; and at the period of his admission he laboured under palsy of the arms and hands. Blood was drawn from between the shoulders, strong opening medicines were given thrice daily, and a tepid bath was used on alternate days. A week after a blister was applied between the shoulders. This was followed by repeated blisters to the arms, colchicum and camphor mixture were given to relieve wandering pains which were complained of, and the cure was completed with Peruvian balsam.

(185.) A painter, aged 30, of irregular habits, and who had been employed in his trade for fifteen years, suffered under four attacks of colic, the last two of which were accompanied by some dropping of the hands. On the 28th of November, while flatting, he experienced a feeling of tightness and burning across the scrobiculus cordis, followed by pain in the umbilical region, numbness of the shoulders and arms, acute headache and obstinate constipation. The abdomen was fomented, a tepid bath was ordered, and a pill, composed of tartar-emetic and opium, was given every six hours, with occasional aperients. On the 3d of December he felt little if at all better, when twenty leeches were placed on the abdomen, and the aperients and pills were continued. On the day after 30 leeches were again applied, which very much relieved his bowels; but a sense of burning pain was felt about the centre of the sternum, shooting across to the back and increased by inspiration, for which a blister was ordered and three grains of extract of hyosciamus, with two of camphor, were given every six hours with success. Until the 10th he was convalescing, when a "gnawing sensation" was felt between the shoulders and down the arms and legs, which required the application of fifteen leeches. On the 12th the pain in his back was gone, but he felt "weaker than ever." Eight ounces of blood were withdrawn from his nape by glasses on the 12th; as much more on the 13th; and as much again on the 14th; under which treatment he so rapidly improved as to be able to leave the hospital in a few days thereafter.

This case is strongly corroborative of the author's views, as to the implication of the brain and upper part of the spinal cord in saturnine palsy. Although the pain, which had been at first experienced between the shoulders, had been wholly removed by the leeches, still his complaint remained in every other respect unabated, until by repeated cupping the cerebral congestion, which no doubt was the remote cause of the great weakness complained of, was entirely and permanently dissipated. In the 187th case the same principle is perhaps still more forcibly illustrated. A countryman, aged 43, who had always enjoyed good health, came up to town and was employed in a paint-manufacture in grinding and mixing preparations of lead, mercury and arsenic. Seven weeks had elapsed in this occupation when a pinching sensation was felt about the abdomen, accompanied by costiveness and followed by numbness and tremor of his right hand, violent headache, dimness of sight and a copious expectoration of mucus streaked with blood. Cupping on the nape and aperient medicine gave him some relief ; but as his abdomen was very painful twelve ounces were taken by glasses from the scrobiculus cordis and the aperients were continued. During the second day after he was admitted his headache became intense and in the night he was attacked with a fit of violent delirium, which lasted three or four hours ; for which leeches were applied to the temples, a blister to the nape, and five grains of the ext. hyosciam. were given every four hours. On the next day he was much better, but his head was still afflicted by shooting pains, and until he was dismissed cured he was occasionally subject not only to them, but to wanderings of intellect and general agitation. The last case, which we shall record, was treated by strychnia, and although its exhibition was long continued before any very palpable benefit was produced, yet the Doctor thinks that its employment was on the whole productive of good.

(186.) A middle-aged man, who had followed the occupation of painter for twenty years, and had experienced during that period three rather sharp fits of painter's palsy, came under the Doctor's care, on the 4th of November, with rigors, obstinate costiveness, and pain and weakness in the arms, with a general feeling of debility. Opening medicine was given, a blister was applied to the elbow (right ?) and splints were placed along his arms and hands. On the 8th little change was visible, when the twentieth part of a grain of strychnia, dissolved in alcohol, was applied to the blistered part over the extensor muscles of the (right ?) fore-arm. On the 10th, the quantity of strychnia employed was increased to the tenth part of a grain; on the 11th, to the 8th ; on the 12th to the 6th; on the 16th to the 4th, and on the 17th to the 3d of a grain. A considerable degree of pain and some spasmodic starting of the affected muscles soon succeeded each application of the strychnia, and on the 13th the arms were "decidedly relieved." One-third of a grain was applied almost daily from the 17th of November till the 15th of December, with the effect of producing very marked spasms, and rendering the right arm, which was at first the weaker, much the stronger of the two. On the 15th of December erysipelas of the face came on, and the nux vomica was not afterwards employed; but the patient slowly improved, and after some weeks left the hospital much better, yet still weak.

From these cases the reader will be able to make himself tolerably familiar with the mode of treatment which Dr. Bright employs in this disease. It appears to us that the good effects of bleeding from the nape are rendered so obvious by them that, however physiologically difficult it may be to associate the local paralysis with the cerebral disturbance, or to explain in what way the relief of the latter can promote the removal of the former, it will be of great practical importance to keep in view this connexion. It would, unquestionably, be desirable to ascertain how far the pathology of the brain and cord in such cases countenances this theory; but we are so far persuaded of its plausibility from the evidence before us, that we anticipate with tolerable confidence its support by future observation. It has been usual since the days of Darwin to combat the spasmodic pains, which so usually accompany the intestinal apathy in palsy from lead, with large doses of opium, and this practice has been sanctioned by its success ; but on referring to the work before us, it will be seen that this medicine has not been very largely employed, and never save in connexion with opening medicines. If congestion of the brain or cord have any thing to do with the production of either the paralytic or spasmodic symptoms of this affection, it is obvious that the removal of such a state will be more effectually promoted by local depletion than antispasmodics, and that the use of opium must be limited to the counteraction of an effect, rather than to the dissipation of the cause. The warm bath, fomentations and poultices will tend to allay the abdominal irritation; and blisters to stimulate the palsied members with splints to support them should not be neglected. Purgatives are required to overcome the torpor of the intestinal tube, and it has been customary to administer them in large doses; but Dr. Bright cautions us against such treatment in the following terms—

"Calomel and other purgatives should be used with some caution, and not continued after the actual necessity for their exhibition has ceased, as the large intestines are in a peculiar and a feeble condition, and not well able to resist severe irritation; so that I have undoubtedly seen, extensive destruction of the mucous membrane of the colon, induced in this disease, by the injudicious use of purgatives: and it is by keeping down the tendency to inflammation that leeches applied to the abdomen, and sometimes to the anus, are found of great service, not only giving relief to the pain, but facilitating the action of purgatives.

The diseased condition of the mucous membrane of the colon in cases of this kind deserves well to be borne in mind: the frequent irritation to which the viscus is exposed appears gradually to thicken the cellular substance beneath its mucous membrane, so that the surface becomes thrown up into ridges, thick, hard, and unyielding; and the membrane itself seems to lose part of its power of resisting injury; it becomes abraded, and is discoloured by the fæces, and covered with shreds, probably in part derived from the separating and sloughing of the membrane itself, and partly from the effusion of lymph.—It is not only in colic from lead that this condition of the colon exists, but I have seen it take place in other cases of obstinate or habitual constipation; particularly after the employment of much purgative medicine." 393.

It was once believed that *lead* was the only mineral, which could induce palsy; but a more perfect acquaintance with these substances has demonstrated the fallacy of this opinion. It is not improbable but that most of the mineral poisons, if taken in small quantities and for a sufficient length of time, might act upon the nervous system in a way somewhat analogous to that now described, as we have reason to believe that most of them convey their baneful influence into the constitution through the medium of the nerves. The Doctor has met with some instances illustrative of the agency of mercury in this respect, when long permitted to operate upon the system; but the symptoms which it occasions are in many points different from such as characterize the agency of lead.

(243.) A water-gilder, aged 31, of intemperate habits, who had already suffered an attack of palsy in consequence of his occupation, was admitted into hospital labouring under headache, soreness of the gums, and an involuntary convulsive motion of his hands. "If he attempted to make any voluntary exertion, as taking hold of any thing, the hand was thrown in every direction with short but violent convulsive catches. When lying quite undisturbed the motion was often for a time suspended; he had also a hurried, convulsive, and indistinct mode of articulation—the left (hand) was most convulsed and the moment he was spoken to the convulsive action increased." After his bowels had been well opened with castor oil, to which a few drops of laudanum were added, he was put upon low diet and beef tea, and ordered one grain of the sulphate of zinc three times daily, with the subcarbonate of ammonia julep. Three days afterwards the zinc was increased to two, again to three, and ultimately to eight-grain doses. At first a burning sensation was experienced in the hand most affected, together with some head-ache and vertigo; but the tongue cleaned, the pulse came down, the spasmodic action grew less violent, the speech improved and, after more than three weeks' employment of the zinc in the doses just mentioned, he left the hospital almost well.

(241 and 2.) A man and his wife applied for assistance at Guy's Hospital with the following symptoms. They were emaciated and sallow, unable to stand steadily or speak distinctly, and they were excessively weak. Their gums were ulcerated and their teeth loose, the least mental emotion aggravated every symptom, and when ordered to grasp any thing with their hands it was obvious by their movements that they were to a certain degree independent of the will. This couple were in the habit of supporting themselves by extracting mercury, by means of pressure, out of the leathern bags in which it is imported; and as their dwelling was not only their sitting apartment during day, but also their bed-room and their workshop, its air was constantly impregnated with this mineral. The woman lost most of her teeth, and, worn out by constant irritation, died. The man was removed from this impure atmosphere, took stimulants and tonics and recovered.

"The cases which I have thus witnessed (says the Doctor) present a considerable variety in the mode by which the poisonous influence of the mercury has been communicated; but in all, the atmosphere was probably impregnated in a very high degree. The symptoms as well as the general treatment which appears to have most power in controlling them, point out some analogy and connexion between this disease, and chorea; and though there is something quite peculiar in the character of the agitation, and the quick spasmodic catches, which are not to be mistaken, in the palsy from mercury, yet the actions are spasmodic as in chorea, and, as in that disease, they affect the muscles of the extremities and the power of articulation, and, as in chorea, they are rendered more and more severe, the more the mind is agitated: but there is this difference, that in chorea the irregular motions are often for a short time under the control of the will. With respect to remedies, a due regulation of the bowels, and the administration of tonics appear the most efficacious in both diseases. In chorea likewise, if we can discover the exciting cause, we strive to remove it; but in the palsy from mercury, the exciting cause being obvious, we are more directly led to its removal as a chief indication in the treatment." 499.

There still remains one other cause of cerebral pressure, or rather one other class of causes, with a brief reference to which we shall close the present article. When the brain or its membranes are injured by external violence, such as by blows or falls, symptoms are not unfrequently occasioned which strongly resemble those that result from ordinary apoplexy. Sometimes the mental functions are instantaneously extinguished or eclipsed, the stomach soon discharges its contents, then a state of sopor supervenes out of which the patient can be roused with difficulty, and after a few hours or a few days every unfavourable symptom gradually subsides. In other instances symptoms of cerebral irritation, if not of actual inflammation, succeed to the period of collapse, and violent delirium, convulsions, palsy and apoplectic coma precede death. In cases marked by the former set of phenomena, the brain is considered to have suffered *concussion*; when the latter are observed phrenitis, or meningitis, or both are apprehended. Concussion is, however, equally formidable in its effects with actual inflammation; and it becomes a question of some interest to discover the immediate state of brain attendant on this affection. In cases of death from concussion almost the only appearances, it would seem, are minute lacerations of the brain and its membranes, either upon its surface or within its substance. When such injuries can be detected there can be no difficulty in accounting for death; but where no such appearances nor indeed any vestiges of structural injury are perceptible after the most attentive autopsy, it must be acknowledged that it is more easy to conjecture than to demonstrate the proximate cause. No doubt an organ, so delicately organized as the human brain, may receive even textural violence without betraying it to the rude inquest of the scalpel; and it is equally capable of belief that functions, at the same time so essential to life and so easily deranged, as those which this organ is destined to discharge, may be suddenly and for ever arrested by an act of violence, not rude enough to stamp its impress in such palpable characters as to be deciphered by the eye of the anatomist. Therefore, it has been usual in all such cases to fancy the existence of what we cannot see, and to regulate our treatment on the presumption that, if the brain or its appendages be not actually disorganized, they have been so violated as to demand from us the same attentive, prompt and energetic management. Symptoms of *pressure from accidents* may depend upon fracture of the skull and depression of the fractured bone—upon detachment, or laceration of the dura mater—upon effused blood between it and the calvarium, or between the arachnoid and it—upon laceration of the arachnoid and pia—upon the effusion of blood between these membranes and brain—upon laceration of the cineritious or medullary, or of both these portions of the cerebral tissue; and, lastly, upon the extravasation of blood into any of its ventricles. As the bare enumeration of these different effects of external injury is all that we can pretend to in the present place, more especially as they are purely surgical subjects, we shall merely extract one or two of the most interesting histories, which the Doctor has brought forward, to illustrate the effects of pressure upon the spinal cord.

(197.) About six weeks before his admission into hospital, W. B., aged 20, experienced some pain at the upper part of his neck, accompanied by stiffness, but without knowing from what cause, and when admitted his head was bent forward upon his chest, he complained of pain when the cervical vertebræ were pressed, there was an apparent displacement of their spinous processes, and his right side was almost completely hemiplegic. Leeches and other local measures were at first employed; but his neck still fell forward and his step was palsied. A seton was then inserted into his neck, and various remedies were tried; but he became obviously weaker. In the latter end of November the left leg began to lose its power, but not its sensation, and felt cold ; in January he experienced cold chills, and complained of dyspnœa attended with constriction across the chest; in February erysipelas attacked his face and head, and he became quite unable to move any part of his body ; and without having ever felt the least headache, or head symptom of any kind, he sunk. No disease could be detected in the brain. The processus dentatus of the second vertebra was so enlarged and displaced that the foramen magnum was much contracted, and the spinal cord at this point looked vascular and presented a darker color than natural for the extent of about a pea. In one or two other portions the spinal cord showed evidence of congestion, but it was so slight as not to be admitted by some present.

In this case the displaced dental process seems to have pressed more especially upon the anterior column of the spinal cord, seeing that with the exception of slight numbness and a feeling of cold, the palsied parts were in full possession of their sensibility. In that which follows the same phenomenon is illustrated, but as arising, not from any independence of the motive and sensatory nerves, but from general osseous disease.

(198.) A black sailor was admitted into Guy's with immoveable stiffness of his neck and general rigidity, so that he walked badly, raised his arms with pain, and was perfectly unable to open his mouth. Every plan was taken to relieve him but without effect, and he at last died, having previously lost almost all power of motion, without his faculty of sensation being, however, in the least impaired. "The disease wanted (says the Doctor) some of the characters of paralysis; it was like the stiffness of chronic rheumatism, without any of the swelling." The lungs were full of small, hard tubercles—the skull-cap was thick and remarkably heavy—the arachnoid was somewhat thickened and opaque—the brain was firm, but rather exsanguine—both the occipital and dental articulations of the atlas were diseased—the processus dentatus was also diseased, and slightly displaced—the rest of the cervical vertebræ were anchylosed—the lower jaw was also immoveably attached to the upper jaw at both articulations.

By an unfortunate oversight the other joints of this subject were not examined ; but, from the rigidity in his motions during life not having been confined to the neck and jaws, there can be little doubt but that the osseous disease was much more extensive, if not general. Having, with these remarks, arrived at the Doctor's third section, on *Diseases of Irritation* connected with the nervous system, to which the whole of his *second* volume is appropriated, we must again part with these *Reports* for the present, leaving for the next number our third and last article on this most practical and increasingly interesting performance. Till then we shall refrain from alluding to one or two points, connected with the diseases now described, which merit the attention of the reader ; and also from stating, more fully than has been already done, the very high esteem in which we hold Dr. Bright as a scientific pathologist and practical physician.

